# The Waste Products of the Body

**Published:** 1890-01

**Authors:** Charles Mayr

**Affiliations:** Springfield, Mass.


### THE WASTE PRODUCTS OF THE BODY.[1]

BY PROFESSOR CHARLES MAYR, SPRINGFIELD, MASS.

THE chief instruments supposed to be used in a dental office are excavators, pluggers, and forceps, to which may be added vulcanizers, and such appliances; but the chemical "tools" have also been multiplying of late too. The various antiseptics have come in use more and more; anæsthetics and disinfectants, which constitute a large proportion of the *materia medica* of dentists' use at present, are what might be called of a defensive nature. Very little of it is positive; that is, almost all medicines used are aimed at the destruction of some enemy, not at the strengthening of the resisting body in the struggle for life which goes on and in every cell, every aggregation of cells, every tooth, every individual, every collection of individuals, every thought, and every train of thoughts, every system of philosophy or creed. This struggle for life can be essentially reduced to two contending bodies,—the one the aggressor, and the other the defendant. As in warfare, between the large collection of cells called man the survival of one party is determined by its own strength or by the weakness of the other party. We have in history very many illustrations where a nation has been victorious in its struggle against another nation by its superior strength. Of course one has to be the weaker, but both may be above the average in strength, and yet the still more vigorous party will be victorious. We all want to be conquerors, none of us want

---

[1] Read before the New Jersey State Dental Society, at its nineteenth annual session, at Asbury Park, July 19, 1889.

to be beaten and crowded out; for nature has only one penalty for being beaten, and that is death. The conqueror sooner or later must die. The conquered old Greeks and Romans are dead. Certain elements among them which were stronger than those of the conquering barbarians have survived. A conquest does not always mean a conquest in every particular. The Goths, in the third, fourth, and fifth centuries, conquered the whole of Spain. They conquered the men, but they did not conquer the women. The Spanish women taught the children of the Goths their unconquered language, and thus the conqueror was vanquished on the field in which he was weak. The Spanish woman and her language utterly annihilated the Goths. There are not a hundred words in the Spanish language to-day which testify to the possession of that country by the Germanic invaders for three hundred years, and, if we did not know the events from history, no linguist would ever suspect such a long and thorough dominion.

The same principle applies to every single cell in the individual man. Every man commands an army of thousands of millions of soldiers. The enemy is just as numerous. Each cell has its enemy, each aggregate of cells has an aggregate of cells as an enemy, and the aggregate man himself has another aggregate man as an enemy. What is the aim of man's existence? Certain cells of the brain may have different aims, but man, as a whole, wants to live as long as possible and preserve the aggregate of its army intact as long as possible. By two methods he tries to reach this aim: by weakening the hosts and groups of enemies, and by strengthening his own army and groups of soldiers. His staff of generals—that is, his brain—must equal those pitted against him. His soldiers—that is, his cells—must be equal or stronger than those of the enemy. The generals in the brain employ auxiliary troops to strengthen their army. The dentist is one of these auxiliaries. He has studied certain points about the enemy; he has been a spy in their camp. Under the guidance of that Pinkerton of detectives, Dr. Miller, of Berlin, he has traced the enemy into his lair; he has investigated his method of growth and attempted to reach him in a vital spot. Almost all the efforts of dentists have now been directed towards weakening the enemy. What is the use of a weak enemy if it puts a premium on our own weakness? I think it would be much better to have a strong enemy pitted against our own strength.

To carry out the comparison still further, during our youth we build our fortress on which we have to depend after our growth is finished, until, with an army of crippled, maimed, and exhausted veterans, we have to surrender. Our lungs lose daily some of their best cell soldiers. Our heart burns up every day a few of the most active workers. It is, therefore, best to start with as powerful an organization as possible. The special branch of the army which we have to consider in this meeting is that of the masticators, a branch of the sutlery department. Now, many a strong army has been beaten in its sutlery department. They do not have to do any active service; they have to attend to, perhaps, less heroic wants than the brain or the eyes, but without strong grinders there cannot be a strong army. The building up of strong teeth in children seems to me to be a far more important problem than the extracting of six-year molars. It is true that General Brain has also provided for that emergency. It has hired steam grinders and furnishes the grinding of the sutlery department. It supplies in hash-shape all vegetables, meats, and cereals. Bread and cheese are made with mushy softness. But without strong masticatory organs we will always be conscious of a weak department in our army. The best and only natural process of growing good teeth in children is to have their parents grow good teeth, of course before the children are born; which saying is not quite as ridiculous as it might at first appear, for it may be observed that parents very frequently do things for the good of their children which they ought to have done before the children were born. But the fact remains, in spite of all those probably correct principles, that children are born with weak teeth. What shall we do? We cannot afford to kill them and grow better ones. For some time it has been the peculiar treatment of dentists to give phosphate of lime and phosphate food in general, with the idea of supplying that which was supposed to be wanting,—the lime salts in teeth. You can pack such children in a lime-barrel, and their teeth will not take up a particle more. You can feed them lime hash and lime stew without effect. The lime has to be introduced through the proper channels and in proper form. The digestive department of the body is just as full of red tape as that of any government. All its supplies have to go a certain regulated course, without which they are not accepted.

Another group attached to the staff of humanity has made the investigation of leaks in the various departments their specialty. Chemists and physiologists belong to that group. Very often they are not admitted to the interior workings of the various departments, but they examine the refuse and see what is going on. They also examine the refuse of the body and try to make conclusions about the processes inside.

The urine is a most interesting waste product. As a rule, I do not think it has been considered to come within the province of the dentist; but perhaps I will be able to convince you that it is very important that he should know something about it. Urine contains a certain quantity of solid substances; the rest is water. The solid matter consists of more than twenty thousand different substances. There is salt, urea, phosphoric acid, lime, potash, soda, magnesia, uric acid, etc.

In order to make a perfect analysis of the urine it is necessary to have a very nice set of chemicals. I have come to certain conclusions in my work, which those of you who are given to that kind of work may be able to appreciate.

If we take urine voided at different times of the day, it shows variations in its specific gravity,—say 1021 in the morning, 1018 at noon, 1029 in the evening, and 1030 before going to bed. That is about the regular course. The amount of urea will show a similar variation. The phosphoric acid will show a corresponding variation, and the salts and uric acid also. There is a law which, strange to say, I have not found laid down in any of the text-books, namely, the relative proportion of those constituents in normal urine is almost a fixed one, regardless of the times.

The most constant relation is that of the urea to the phosphoric acid, which in healthy, normal urine is 100 to 8. If 100 parts of urea are excreted, 8 parts of phosphoric acid have to be excreted. The variation of these constituents in normal urine are from a maximum of 100 to 8½ to 100 to 7½. If this relation of urea to phosphoric acid varies any more than that it shows defective nutrition.

I find that the ratio of phosphoric acid excreted is about the same in food. In the body itself the phosphoric acid bears to the nitrogeneous tissue the relation of 10 to 100. If the ratio of free phosphoric acid in the urine is more than 8 to 100, it shows very plainly that the person is living on his body, and not on the food taken. If the ratio is 10 to 100, it is a very dangerous condition. I have found it 11 to 100 during the last days preceding death. If the ratio sinks to 4 to 100, it is a sign that the person is strongly assimilating and gaining in weight. So also does the ratio of urea to phosphoric acid tell us what the state of health of the person is, and is most interesting in that respect.

Dentists have speculated a good deal about the dissolving of phosphates. I have, unfortunately, not been conversant with any specimens from dentists in marked cases to show in what way it would have a bearing. I should say that if the ratio of phosphoric acid to urea is 100 to 9, no phosphoric acid could be assimilated in the body; and if the ratio be 100 to 4, phosphoric acid and tissues would be freely retained in the body.

Another important constituent is uric acid. That is found in small crystals, which look like Connecticut red sandstone. This uric acid, when perfectly purified, is colorless. We do not know where it is manufactured; but we know it bears a close relation to the digestive process. Uric acid in the proportion of 100 to 3½ shows a strong digestion; in the proportion of 100 to 2 it shows a weak digestion; 100 to 3 is about normal. If you find a high amount of phosphoric acid excreted from the body, with low per cent. of uric acid, the person is feeding on his body. If you observe a low per cent. of phosphoric and uric acid, there is a little wasting away of the tissue, and not very good health.

Another important thing is the sum total of the other constituents. Salt occurs in the urine in variable quantities. It depends upon the food taken. After breakfast the quantity excreted is considerably higher than after supper. It goes through the body without much change; therefore, chemically, the ratio of salt is not very important. Of the other constituents there are several thousand in normal urine that bear a strict relation to the unit of urea. Those other constituents ought to be about as 100 to 40–70; 100 to 120 indicates the formation in the body of abnormal products. There are, furthermore, coloring matters. These are totally unknown substances that we have not yet isolated. There are organic substances, like creatinine, etc.; and finally a small quantity of sugar, which is rarely absent. From an examination of the comparative quantities of these constituents you can form an excellent opinion of the working of the body; better than by any other investigation.

I think the subject of urinary analysis is worthy the attention of dentists. Suppose we have found, after investigation, that the per cent. of phosphoric acid in the urine is high; the person is excreting more, in spite of a good digestion, than he ought to; he is consuming more of his flesh than he ought to. What can we do? Shall we give him acid phosphates? Not at all. The acid phosphate is not the phosphoric acid of the body. It is as different from that of the body as it is from sulphuric acid. Therefore the idea of prescribing phosphate drinks for the purpose of supplying the deficiency of the phosphates seems to me unreasonable and useless. Bone phosphate, made from the powdered bones of animals, was given, in olden times, in the shape of powdered mice, carefully dried, etc. A little bit of that phosphate may possibly be assimilated, but I have my doubts whether any perceptible particle can be assimilated. The assimilating apparatus will take only things that are presented in a certain prescribed form; it will receive the mineral elements when presented in combination with albuminous substances, as in bones, plants, flesh tissue, etc., but it will not accept phosphates alone. So I think that in cases of excessive excretion of phosphoric acid it is of no use to try to put more into the body; it does not do any good. We have to see whether that excessive excretion has not something to do with wrong digestion. As a rule, where there is an excess of phosphoric acid excreted you will find low uric acid, showing that, no matter how much phosphates you put into the body, they are not assimilated. We have to dircet our attention to the digestive process. I am not sufficiently conversant with the methods which you use to correct or improve them.

If a child were to be fed exclusively on potatoes, there might be a certain deficiency of phosphates in the body; and I do not doubt that, to a certain extent, a lack of hardness in the teeth of the child might be ascribed to the defective food.

It has been found that a certain ratio must be preserved between urea and phosphoric acid, in order to keep the proper balance of the economy of the body.

This conclusion, which I have reached by comparison of thousands of experiments, seems to me a very valuable one. By examining the urine of a patient whose teeth seem to decay and go rapidly, something valuable might be found, in order to see whether this is owing to a solution of the phosphate substance within the body or to the action of external agencies. This might be of special value in children. Now, perhaps having discovered such a leak, what can we do? We have to examine whether the wastefulness in this department is owing to incompetency or to lack of supply. If the first is the case, as we cannot do away with incompetent intestines, we must try to make them more competent by either vigorously arousing their laziness with what we call tonics or by gently pleasant treatment in the form of rest. The treatment has to be adjusted to the individual. If, on the other hand, we are well satisfied that there is a deficiency of supply, we have excellent and abundant supplies. Flour of the entire wheat, rye, and oats contain a vast amount of phosphates, and in a form in which they are combined with albuminous substance, and accepted by the red-tape department of the body. If they are not combined with albuminous substances, they are most universally rejected. Another very valuable indication as to the requirements of this department may be furnished by another product of waste of the body, namely, uric acid. We do not know exactly from what it is produced, nor what part of the body produces it, but a relation between the digestive powers of an individual and the excretion of uric acid certainly exists. Especially in disease of the kidneys the ratio falls very low, while in those of the stomach it goes high. If we therefore find a deficiency of phosphoric acid excreted with the ratio of uric acid high, we may consider that person as suffering, if he suffers, from too rapid digestive process, a thing just as bad as too slow. If we run a boiler which requires for good working two tons of coal a day with the consumption of four tons, we not only waste money, but we burn out the boiler much quicker. If the excretion of phosphoric acid is normal, while that of uric acid is high, probably only dyspepsia is indicated,—that is, irritability of the stomach. If phosphoric acid be normal and uric acid low, the person is in danger of having to live on his body at the least disturbance of digestion. If, on the other hand, phosphoric acid is high and uric acid is low, that person is severely sick. He lives on his body and assimilates but little.

Thus you will see how those two constituents alone tell a pretty fair story of the working of the sutlery department of the body, and if a body lives on its own phosphates, both will suffer more or less; with the phosphates there go also a number of cells and cellular substance that ought to be used otherwise than for anthropophagy.

As you will see, I don't take any great stock in the various forms of acid phosphates, the phosphate drinks, etc., which produce as their most important result strong plethora in the pocket-book of makers and corresponding pecuniary depletion in the consumer, which, furthermore, tax the kidneys which have already plenty to do with the unnecessary call for earning money for a patent medicine maker.

